# A Multidisciplinary Approach to Silent Ischaemic Strokes Unveiling Primary CNS Vasculitis: Exploring Beyond the Apparent

**DOI:** 10.7759/cureus.94547

**Published:** 2025-10-14

**Authors:** Syed Muhammad Meeran Hussain, Anum Faisal

**Affiliations:** 1 Medicine, Frimley Health NHS Foundation Trust, Frimley, GBR; 2 Stroke Medicine, Frimley Health NHS Foundation Trust, Frimley, GBR

**Keywords:** cns vasculitis, ischaemic stroke, multidisciplinary approach, silent infarcts, young female

## Abstract

Diagnosing first-episode strokes due to central nervous system (CNS) vasculitis is challenging, particularly when systemic vasculitis features are absent. We present the case of a 42-year-old female patient with hypertension, type 2 diabetes mellitus, and a psychiatric history, who presented with confusion and sleep disturbances. Initial CT brain revealed multiple hypodense foci suggestive of vascular insult, and MRI demonstrated multiple ischaemic infarcts in the right middle cerebral artery (MCA) territory, with features raising suspicion for medium-vessel vasculitis. Despite dual antiplatelet therapy (DAPT), she experienced further silent infarcts in the left MCA territory. A multidisciplinary evaluation, including stroke, neuroradiology, and rheumatology specialists, led to a diagnosis of CNS vasculitis based on neuroimaging findings, elevated cerebrospinal fluid (CSF) protein, negative autoimmune screen, and normal complement levels. She received intravenous methylprednisolone followed by oral prednisolone and methotrexate. Primary CNS vasculitis (PCNSV) should be considered in young patients with unexplained recurrent or silent ischaemic strokes, and a multidisciplinary approach is essential for timely diagnosis and management.

## Introduction

Central nervous system (CNS) vasculitis is a rare, potentially life-threatening inflammatory disorder affecting cerebral blood vessels, characterized by the absence of systemic involvement. The estimated incidence is approximately 2.4 cases per million per year [1,2]. Clinical presentation is highly variable, including headache, cognitive dysfunction, focal neurological deficits, seizures, and ischaemic stroke [3]. Stroke may be the initial manifestation, especially when imaging shows multifocal lesions across different vascular territories. Early recognition and appropriate immunosuppressive therapy are critical to prevent neurological deterioration [4,5].

## Case presentation

A 42-year-old woman with a history of hypertension, type 2 diabetes, and mental health conditions presented with confusion and sleep disturbances. Physical examination revealed cognitive dysfunction without focal motor, sensory, or cranial nerve deficits.

**Figure 1:**
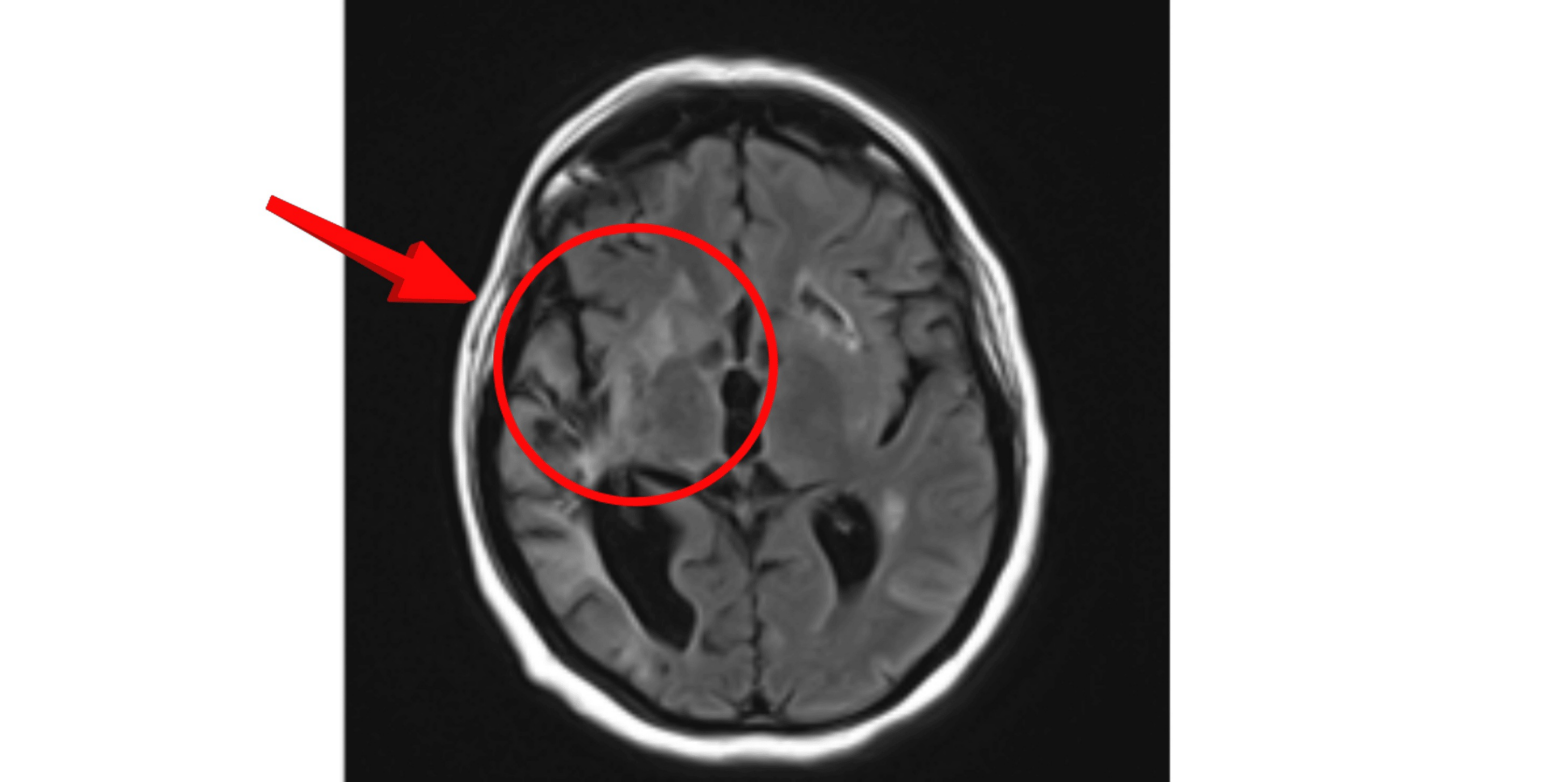
Axial MRI brain showing scattered hyperintense lesions in R MCA territory MRI: magnetic resonance imaging; R MCA: right middle cerebral artery

Computed tomography (CT) of the head demonstrated multiple hypodense areas, suggestive of vascular insult. Subsequently, she was started on dual antiplatelet therapy (DAPT) following the acute stroke protocol, and her vascular risk factors were managed. MRI head further revealed scattered hyperintense lesions in the right middle cerebral artery (MCA) territory, suggestive of infarcts (Figure 1).

**Figure 2:**
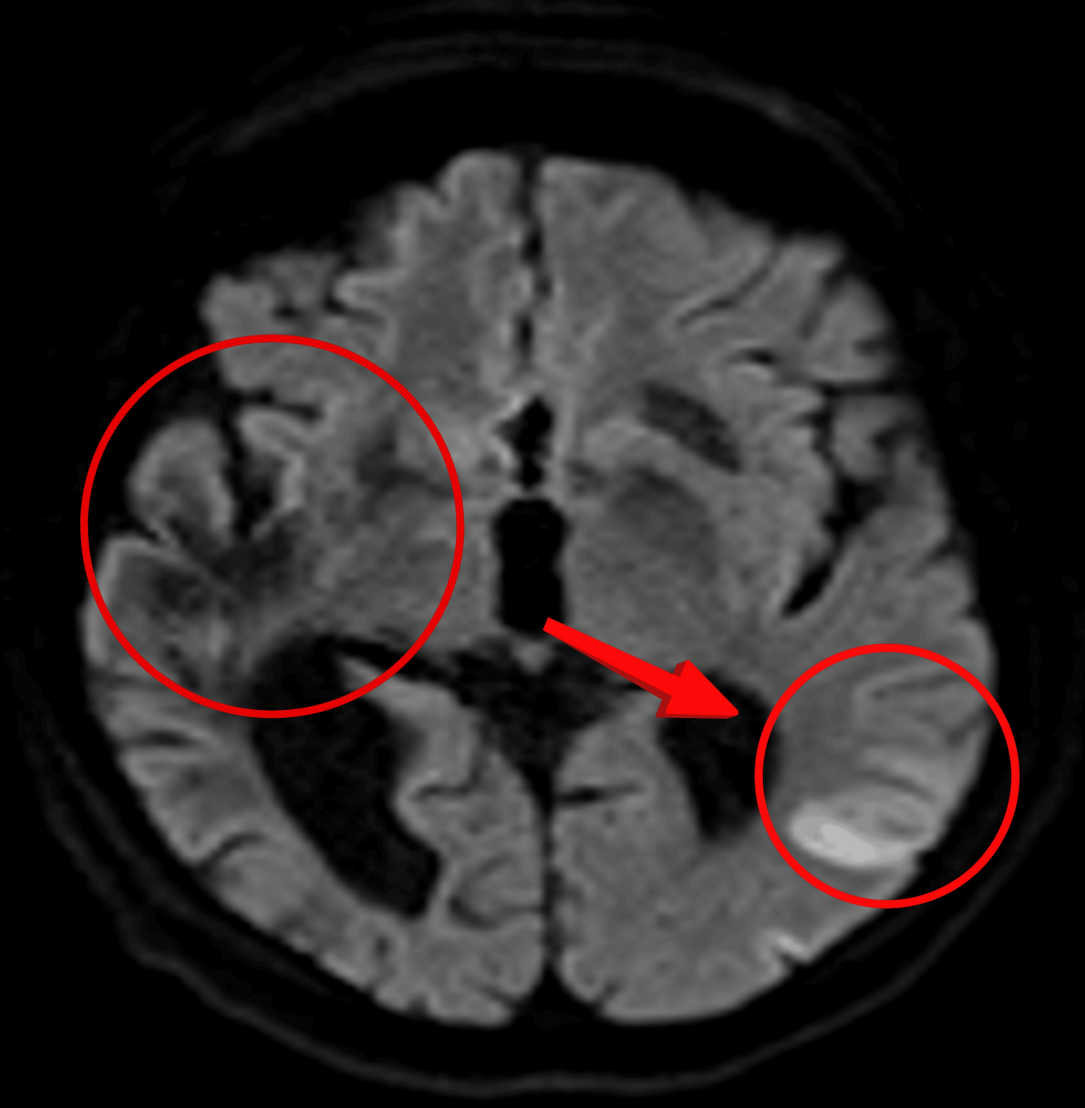
MRI head showing acute infarct in the left MCA territory and an old infarct in the right MCA territory MRI: magnetic resonance imaging; MCA: middle cerebral artery

While in stroke rehabilitation, the patient suffered further episodes of silent ischaemic infarction in the territory of the left MCA, despite being on optimal therapy (Figure 2).

**Figure 3:**
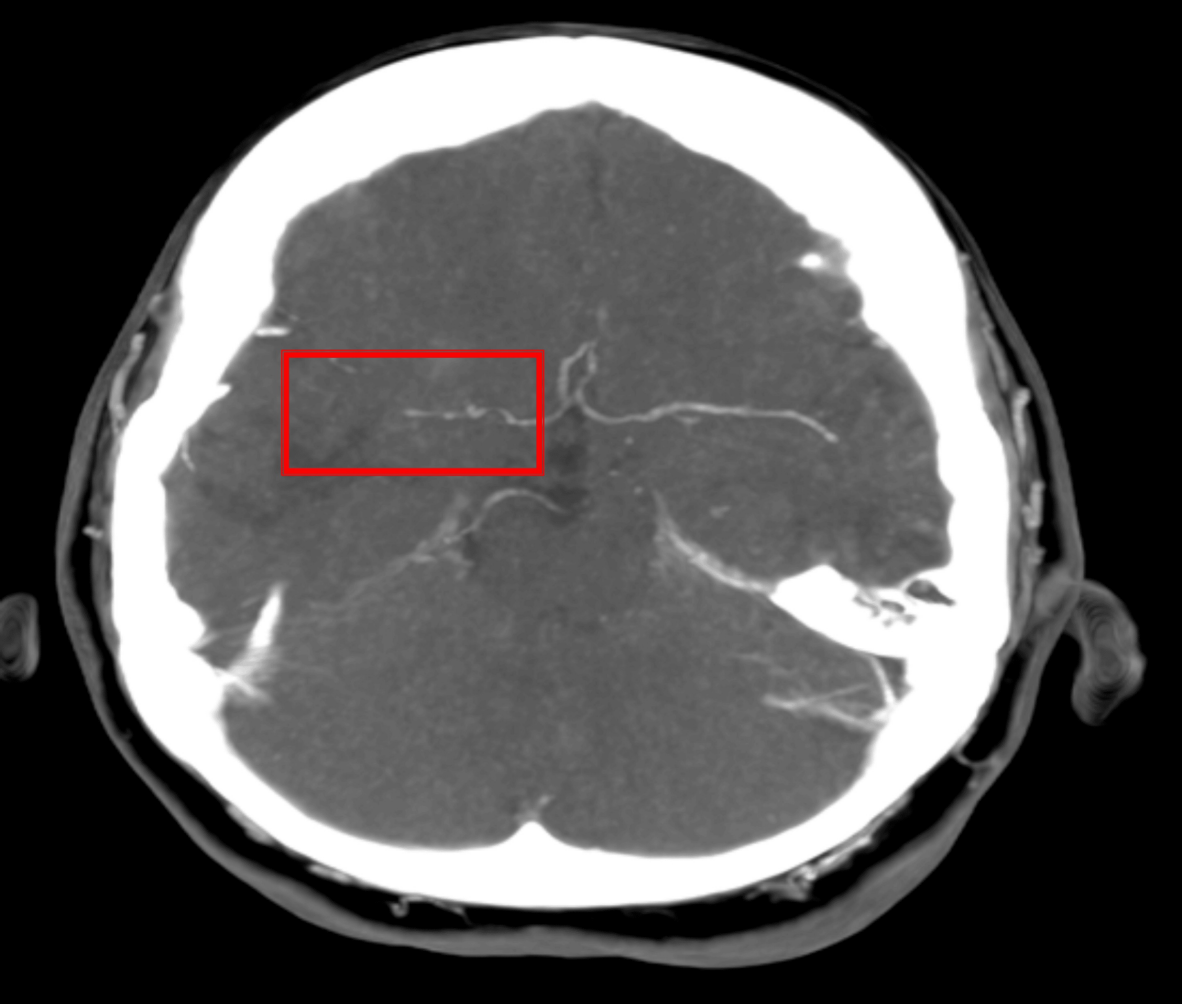
CT Angiogram showing segmental narrowing of the right MCA CT: computed tomography; MCA: middle cerebral artery

CT angiogram was done, which demonstrated a tortuous right MCA suggestive of vasculitis (Figure 3). Cerebrospinal fluid (CSF) analysis revealed elevated protein. Autoimmune testing was negative, and complement levels were within normal limits.

Hence, a multidisciplinary discussion involving a stroke consultant, neuroradiologist, and rheumatologist was held to evaluate her case, and she was diagnosed to have CNS vasculitis on the basis of brain imaging findings, elevated protein in CSF, negative autoimmune screen, and normal complement levels [3,4].

After the multidisciplinary team discussion, she received induction therapy with intravenous methylprednisolone (1 g daily for five days), followed by oral prednisolone (30 mg/day) and weekly methotrexate (10 mg) as a steroid-sparing agent. This regimen reflects established practice, where pulse corticosteroids are combined with immunosuppressants in severe disease [6]. She tolerated the treatment well and was discharged with outpatient follow-up.

## Discussion

Primary CNS vasculitis (PCNSV) is a rare and diagnostically challenging disorder due to its heterogeneous presentation and the absence of specific systemic markers. Clinical manifestations can include headache, cognitive impairment, focal neurological deficits, seizures, and stroke, particularly when MRI reveals multifocal infarcts across different vascular territories [1,2]. Such imaging findings may serve as the first indication of an underlying vasculitic process, especially in patients without systemic inflammatory features [2].

In this patient, the absence of systemic signs, a negative autoimmune screen, and normal complement levels made diagnosis difficult. Elevated CSF protein provided a critical clue, as CSF abnormalities, including mild pleocytosis or elevated protein, are frequently observed in PCNSV and support the diagnosis when systemic inflammatory markers are non-revealing [1,3].

Angiographic imaging can further aid in diagnosis. Computed tomography angiography (CTA) or digital subtraction angiography (DSA) may demonstrate segmental narrowing, vessel tortuosity, or “beading” of medium-sized intracranial arteries [4]. However, such findings are not entirely specific and may overlap with reversible cerebral vasoconstriction syndrome or intracranial atherosclerosis [7]. In this case, CT angiography revealed tortuosity and segmental narrowing of the right MCA, while recurrent silent infarcts on MRI reinforced suspicion of a vasculitic process.

Although brain or meningeal biopsy remains the diagnostic gold standard, sensitivity is limited by patchy vessel involvement [3,5]. Therefore, diagnosis often relies on a multidisciplinary assessment that integrates clinical presentation, neuroimaging, angiographic findings, and CSF analysis [3,5]. Such an approach facilitates the timely initiation of immunosuppressive therapy while minimizing unnecessary invasive procedures.

Treatment aims at rapid suppression of vascular inflammation to prevent further ischaemic injury. High-dose corticosteroids remain the mainstay of therapy, frequently combined with immunosuppressants such as cyclophosphamide or methotrexate for induction or maintenance [6]. Observational data indicate that combination therapy reduces relapse rates compared to corticosteroids alone [6]. In refractory or relapsing cases, rituximab has been employed successfully, underscoring the value of individualized, multidisciplinary treatment strategies [8].

This case highlights the importance of early recognition and prompt management. Recurrent or silent ischaemic infarcts in younger patients, even with traditional vascular risk factors, should raise suspicion for PCNSV, particularly when systemic inflammatory markers are negative [9]. Multidisciplinary collaboration, integrating stroke neurology, neuroradiology, and rheumatology, was pivotal in establishing the diagnosis and guiding immunosuppressive therapy, ultimately improving patient outcomes [2,6].

## Conclusions

PCNSV should be considered in younger or middle-aged patients with unexplained, recurrent, or silent ischaemic strokes, particularly when imaging reveals multifocal infarcts and CSF findings are supportive but systemic markers are non-revealing. A multidisciplinary diagnostic approach and prompt initiation of immunosuppressive therapy are pivotal to improve outcomes.
